# Enzymatic hydrolysis of almond hulls for cultivation of edible filamentous fungi

**DOI:** 10.1186/s40643-025-00940-2

**Published:** 2025-09-29

**Authors:** Lin Cao, Allan Chio, Hamed M. El Mashad, Zhongli Pan, Ruihong Zhang

**Affiliations:** 1 Department of Biological and Agricultural Engineering, University of California, Davis, Davis, CA 95616 USA; 2 Department of Agricultural Engineering, Mansoura University, P.O. Box 35516, EI Mansoura, Egypt

**Keywords:** Almond hull, Enzymatic hydrolysis, Sugar recovery, Liquefaction, Filamentous fungi, Submerged cultivation

## Abstract

Enzymatic hydrolysis and liquefaction of the residual almond hull solids (RAHS) after sugar extraction were studied. The hydrolysis performance of three different enzymes (Cellic CTec2, Viscozyme L and Pectinex Ultra SPL) were investigated. The ability of *Aspergillus awamori* and *Aspergillus oryzae* in utilizing the hydrolysate was evaluated. In 50 mL Falcon tubes, the combination of 200 $\:{\upmu\:}$L/g RAHS of Cellic Ctec2 and 60 $\:{\upmu\:}$L/g RAHS of Viscozyme L enzymes resulted in the optimal performance, with total sugar yield of 47.21%, total fiber conversion of 86.01%, and liquefaction efficiency of 51.61%. Applying these optimal conditions in 250 mL glass bottles resulted in higher liquefaction efficiency of 72.53%, while total fiber conversion was same as that in Falcon tubes. Through a combination of sugar extraction and subsequent enzymatic hydrolysis, a total of 0.55 g of sugars could be released per gram of almond hulls (dry basis, d.b.). The cultivation of *Aspergillus awamori* and *Aspergillus oryzae* in hydrolysate for five days produced uniform fungal pellets, with biomass yields of 0.89 g total suspended solids (TSS)/g sugar and 0.43 g TSS/g sugar, respectively. Both fungal strains were able to utilize sugars in the hydrolysate, including galacturonic acid. This research demonstrates that enzymatic hydrolysis of RAHS enables the recovery of additional sugars from almond hulls, thereby increasing the yield of fungal biomass and improving the overall utilization of almond hulls.

**Supplementary Information:**

The online version contains supplementary material available at 10.1186/s40643-025-00940-2.

## Introduction

The global population is projected to reach near 10 billion by 2050, which makes it critical to find sustainable means of producing food (McKenzie and Williams 2015). Some challenges experienced by the current food supply are resource depletion, greenhouse gas emissions, increased agricultural water usage, and the necessity to reduce waste while increasing output (Koukoumaki et al. 2024). One promising solution to these challenges is the utilization of bioprocesses to convert agricultural byproducts or waste into nutritious food products.

Single cell protein (SCP) is cell mass or protein extracted from microorganisms such as fungi, algae and bacteria. SCP has gained a lot of scientific interest because it can be produced in climate-independent conditions while utilizing agricultural byproducts or wastes (Barzee et al. 2022). Compared to algae and bacteria, fungi can utilize a broader range of substrates, thrive under various growth conditions and can be easily harvested due to their filamentous or pellet forms. Filamentous fungal species within the *Aspergillus* genus, like *Aspergillus niger*, *Aspergillus awamori (A. awamori)*, *Aspergillus oryzae (A. oryzae)* and *Aspergillus sojae*, have been extensively used in food fermentation and the production of organic acids, enzymes, and bioactives, contributing significantly to biotechnological applications in the food industry (Ichishima 2016; Park et al. 2017; Vivek and Venkitasamy 2023). Both *A. awamori* and *A. oryzae* are recognized as Generally Recognized as Safe (GRAS) by the U.S. Food and Drug Administration (FDA) for various food applications, with multiple GRAS notices and regulation codes (e.g., 21 CFR § 184.1685 for *A. awamori* and § 137.105, § 173.130 for *A. oryzae*) (U.S. Food & Drug, 2018). These fungi are also promising for food applications due to their favorable nutritional composition. Based on our previous experiments using almond hull extract as substrate, *A. awamori* biomass contained approximately 18.10% crude protein, 2.28% crude fat and 8.60% crude fiber (dry basis, d.b.), while *A. oryzae* biomass contained 25.30% crude protein, 6.30% crude fat, and 14.30% crude fiber (d.b.) (Cao 2023). Such balanced nutritional profiles suggest their potential as multifunctional ingredients with textural and nutritional benefits in broad food applications.

**Fig. 1:**
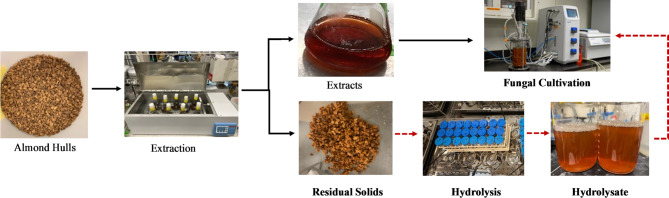
Main processes for producing fungal biomass utilizing almond hulls as feedstock

Almond hulls are significant byproducts of the almond industry. In 2024, the almond industry generated 1.75 million metric tons of almond hulls in California (Almond Board of California 2024). One characteristic of almond hull is its high sugar content, ranging from 26.6 to 56.9% (DePeters et al. 2020). Traditionally, the hulls are used as animal feed or discarded as waste. Recent studies have explored the potential of almond hulls in various applications, highlighting the versatility of these byproducts (Axelrod et al. 2021; Monroe 2021; Najari et al. 2022; Shea et al. 2024). One of the major applications is to extract valuable compounds such as sugars and phenolics from almond hulls by using water or organic solvent mixtures (DePeters et al. 2020; Kiani et al. 2024; Offeman et al. 2015; Prgomet et al. 2019). In our previous research (Cao et al. 2025), soluble sugars were extracted from almond hulls, using hot water, for fungal biomass cultivation. However, nearly half of the hull mass remained as unutilized insoluble solids after water extraction. These residual almond hull solids (RAHS) are rich in cellulose, hemicellulose, pectin, and lignin, and represent a valuable but underexploited resource. Unlike untreated hulls, RAHS contain fewer free sugars, which helps alleviate end-product inhibition often seen during enzymatic hydrolysis (Cardona et al. 2015), making them a more favorable substrate for efficient saccharification. Hydrolyzing these structural carbohydrates not only releases fermentable sugars but also liberates water retained by the pectin matrix, which can hold 10–15 times its own weight in moisture (Holtman et al. 2015). The overall process flow of almond hull valorization through sugar extraction, enzymatic hydrolysis, and fungal cultivation is illustrated in Fig. 1.

Previous research has applied enzymatic hydrolysis to raw almond hulls to produce sugars for biofuel or feed purposes. Holtman et al. (2015) used pectinase directly to raw almond hulls to enhance water and increase recoverable sugar yields for biofuel production by yeast fermentation. However, because the hulls contained high levels of both inherent and hydrolyzed sugars, the overall sugar concentration in the system was evaluated. This led to a great amount of sugar remaining in the liquid trapped within the solids and therefore required several washing cycles to recover sugars from the solids. Sitepu et al. (2023) utilized pectinase to hydrolyze almond hulls and produced yeast biomass through solid-state fermentation for feed applications. However, to the best of our knowledge no studies to date have focused on valorizing the extracted RAHS, which otherwise require disposal or low-value uses. This work fills that gap by proposing a novel strategy for food-related biomass production using agricultural byproducts.

Given the increasing interest in circular food systems and the need to reduce competition with conventional food ingredients, utilizing RAHS provides a low-cost, sustainable, and non-edible carbon source for microbial biomass production. Although enzyme costs are a common concern, this study minimizes dosage to enhance economic feasibility while maximizing sugar recovery. The goal of this study is to optimize the hydrolysis of the residual almond hull solids for filamentous fungal biomass cultivation for food applications. The primary objectives are to select appropriate enzymes and loadings for the hydrolysis of RAHS, scale up the process to produce sufficient hydrolysate for fungal cultivation and evaluate the utilization of RAHS hydrolysate by filamentous fungi. This strategy not only maximizes almond hull usage but also significantly reduces waste and potentially brings additional economic benefits and increases fungal biomass yield for food applications.

## Materials and methods

### Residual almond hull solids preparation and characterization

In our previous study (Cao et al. 2025), soluble sugars were extracted from almond hulls (Independence variety) using hot water and used for fungal cultivation. The extraction process involved soaking ground almond hulls (particle size 2.36–3.38 mm) in preheated deionized water at 80°C for 90 min with a liquid-to-solid ratio of 14. The remaining solids were separated from liquid using cheesecloth and manually squeezed and used in this study as RAHS. After sugar extraction, RAHS was frozen at -20°C until use. The composition of almond hulls and RAHS were analyzed for moisture, neutral detergent fiber (NDF), acid detergent fiber (ADF), lignin and ash contents, by Denele Analytical Inc, using the methods described in Cao et al. (2024). Cellulose content was calculated as the difference between ADF and lignin. Hemicellulose was calculated as the difference between NDF and ADF. Pectin content was determined by measuring the amount of galacturonic acid produced after digesting RAHS samples in a buffered solution with pectinase using modified methods from (Li et al. 2023). Galacturonic acid concentrations were measured using High-Performance Liquid Chromatography (HPLC, Shimadzu SPD-M20A) followed the method described by (Zicari 2016). The HPLC system was equipped with a CBM-20 A controller, an SPD-20 A photodiode array (PDA) detector, and an Aminex HPX-87 H column. Sulfuric acid (0.05 mM) was used as a mobile phase at a flow rate of 0.5 mL/min. The column temperature was controlled at 60°C.

### Enzyme selection and reaction time determination for enzymatic hydrolysis

Three different commercial enzymes were purchased from Sigma-Aldrich. The first enzyme, Cellic CTec2 (C enzyme), is primarily used for the breakdown of complex cellulose materials. It contains cellulase and endo-1,4-$\:{\upbeta\:}$-glucanase, and its activity was measured in our laboratory to be 120 Filter Paper Unit (FPU)/mL using the filter paper assay (Zicari 2016). The C enzyme was employed both individually and in combination with pectinase-containing enzymes, Viscozyme L (V enzyme) and Pectinex Ultra SPL (P enzyme), to explore the potential synergistic effects of different enzymes. Instead of combining multiple single enzymes, the commercially available multi-enzyme formulations were selected and used to provide broad hydrolytic capability suitable for the hydrolysis of RAHS; and simplify process design for commercial applications.

The V enzyme contains a broad spectrum of carbohydrase activities, including pectinase, $\:{\upbeta\:}$-glucanase, hemicellulase and xylanase, which is normally used for the breakdown of plant cell walls and helps in releasing soluble sugars and other valuable components from plant materials. According to vendor data, V enzyme contains 91 FBGU/mL (Fungal Beta- Glucanase per mL), where one FBGU is defined as the amount of enzyme that liberates 1 $\:{\upmu\:}\text{m}\text{o}\text{l}$ of reducing sugars per minute under standard assay conditions. Combo et al. (2012) reported its polygalacturonase and endopolygalacturonase activities of 415.4 U/mL and 11,725 U/mL, respectively, and a protein content of 24.3 mg/mL. P enzyme also contains pectinase, $\:{\upbeta\:}$-glucanase and hemicellulase, which is used for the degradation of pectin and associated polysaccharides in plant materials (Zicari 2016). Its polygalacturonase and endopolygalacturonase activities were reported as 523.8 U/mL and 7,161 U/mL, respectively, with a protein content of 11.8 mg/mL (Combo et al. 2012). The effect of individual pectinase enzymes on the hydrolysis of RAHS was also evaluated. Due to the complexed enzyme compositions of these commercial enzymes, it is impractical to control enzyme loading on activities. Instead, fixed volume-based loadings of 30 $\:{\upmu\:}$L pectinase /g RAHS (d.b.) and 100 $\:{\upmu\:}$L cellulase /g RAHS (d.b.) were used. The loadings were determined based on the optimal levels reported by (Zicari 2016) for sugar beets hydrolysis and were adjusted according to the cellulose, hemicellulose and pectin contents of RAHS. Enzyme loadings for different treatments are listed in Table 1. Blank sample consisted of RAHS only, without the addition of enzymes, were also incubated with the same conditions used in the treatments. The blank served as a baseline level for the sugars and liquefaction, which could indicate the natural properties of RAHS and allow for an accurate assessment of the enzymes’ effectiveness. The initial pH of the hydrolysis system was not adjusted but was measured to be approximately 4.5. pH was monitored throughout the hydrolysis process to evaluate whether it remained within the optimal range for enzymatic activity.

**Table 1:** Loadings of different enzyme mixtures for enzyme selection experiments

Trial ID	Cellic CTec2 ($\:{\upmu\:}$L /g)	Pectinex Ultra SPL ($\:{\upmu\:}$L /g)	Viscozyme L ($\:{\upmu\:}$L /g)
Blk	0	0	0
C	100	0	0
*P*	0	30	0
V	0	0	30
CP	100	30	0
CV	100	0	30
CPV	100	15	15

Note: “Blk” demotes the blank sample with no enzyme added. “C”, “P”, and “V” represent trials using Cellic CTec2, Pectinex Ultra SPL, and Viscozyme L, respectively. Combinations such as “CP”, “CV”, and “CPV” indicate trials where two or three enzymes were used together

All enzymatic hydrolysis experiments were performed in duplicate in 50 mL Falcon tubes with a total mass of RAHS and deionized water of 20 g at a total solid (TS) loading of 10%. All the Falcon tubes were incubated in a shaker incubator at 50°C with a shaking speed of 200 rpm. Samples were collected by removing two tubes from each treatment at time intervals of 4, 8, 12, and 24 h. After collection, the sampled tubes were centrifuged at 5000 rpm for 30 min. The supernatants were then decanted into tared 15 mL Falcon tubes, and their mass was measured and recorded. The collected supernatants were heated at 99°C for 10 min in a water bath to deactivate the enzymes (Zicari 2016). Subsequently, the supernatants were frozen at -20°C for later sugar concentration analysis using HPLC.

The optimal mixture of enzymes and the reaction time for enzymatic hydrolysis were determined by comparing the liquefaction efficiency, total sugar yield, and total recoverable sugar yield. The liquid content in solid phase was determined by measuring the mass reduction of the residual solids after enzymatic hydrolysis and after overnight drying at 105°C (APHA et al., 2017).

The liquefaction efficiency was calculated by dividing the mass of supernatant obtained after centrifugation by the initial total mass, as expressed in Eq. (1):

1 $$\begin{array}{l}\:Liquefaction\:\left( \% \right) = \:\\\frac{{Mass\:of\:supernantant\:after\:centrifugation\:\left( g \right)}}{{Initial\:total\:mass\:\left( g \right)}}\: \times \:\:100\% \end{array}$$

Total sugar yield was calculated by dividing the total mass of soluble sugars in the liquid by the initial dry matter of RAHS, as outlined in Eq. (2). For this calculation, it was assumed that the sugar concentration in the liquid phase in the solid was equivalent to that in the supernatant. Furthermore, the density of the supernatant was assumed to be 1 g/mL.

2 $$\:Total\:sugar\:yield\:\left(\%\right)=\:\frac{{C}_{ss}\times\:\:\left({V}_{s}+{V}_{ls}\right)}{{M}_{RAHS}\times\:1000}\:\times\:\:100\%$$

where $\:{C}_{ss}$ is the sugar concentration in supernatant (g/L); $\:{V}_{s}$ is the volume of supernatant (mL); $\:{V}_{ls}$ is the volume of liquid in solid after hydrolysis (mL); $\:{M}_{RAHS}$ is the dry matter of RAHS (g).

Recoverable sugar yield was defined as the sugar yield in the supernatant, calculated using Eq. (3):

3 $$\:Recoverable\:sugar\:yield\:\left(\%\right)=\:\frac{{C}_{ss}\:\times\:\:{V}_{s}}{{M}_{RAHS}\:\times\:\:1000}\times\:100\%$$

The concentrations of the soluble sugars (glucose, sucrose, xylose, arabinose, galactose, fructose and mannose) were analyzed using HPLC, which followed a method modified from Zicari (2016). Prior to analysis, samples were filtered through 0.22 $\:{\upmu\:}\text{m}$ syringe filters to remove suspended solids. Those sugars were analyzed by HPLC using a RID-10 A refractive index detector with an Aminex HPX-87P column. Milli-Q water was used as the mobile phase at a flow rate of 0.5 mL/min. The column temperature was controlled at 80°C. Glucose, sucrose, xylose, arabinose, and galactose were eluted individually, whereas fructose and mannose were co-eluted and therefore were quantified as a cumulative peak. However, according to the study from Sitepu et al. (2023), mannose levels in almond hull hydrolysate was too low to detect. Therefore, the cumulative peak was assumed to be fructose. Additionally, galacturonic acid was quantified by HPLC as mentioned in the previous section.

### Determination of enzyme loadings

Experiments were designed to evaluate enzymatic hydrolysis using different enzyme loadings. The two enzymes, C and V enzymes, were selected from previous experiment. The C enzyme was tested at five different loadings: 50, 100, 150, 200 and 250 $\:{\upmu\:}$L/g RAHS (d.b.), while V enzyme loadings were 20, 30, 40, 50 and 60 $\:{\upmu\:}$L/g RAHS (d.b.). A two-factor factorial design was employed, and each set of conditions was duplicated. The experiments were conducted in 50 mL Falcon tubes as described in previous section for 24 h. Sugar yields were determined after enzymatic hydrolysis as previously described.

### Large scale enzymatic hydrolysis using optimum enzyme loadings

Large scale enzymatic hydrolysis was conducted in six 250 mL glass bottles, each containing 200 g material at 10% TS loading. This scale-up represented a five-fold increase from the initial 50 mL Falcon tube trials. The optimum enzyme loadings identified in the small-scale Falcon tube tests were applied.

After hydrolysis, the contents from every two glass bottles were transferred and combined into one 500 mL centrifuge bottle. These were then centrifuged at 4°C and 5000 rpm for 30 min. The supernatant was subsequently heated to 99°C for 10 min to deactivate the enzymes. Fine suspended solids were further removed via vacuum filtration using standard filter paper. Liquefaction efficiency and sugar yields were analyzed as previously described and compared to those obtained from the small-scale results.

### Evaluate the fungal growth on RAHS hydrolysate

The total sugar concentrations, total nitrogen (TN) and total organic carbon (TOC) of the hydrolysate from the large-scale hydrolysis were analyzed. Sugars were quantified using HPLC as described earlier. Concentration of TN and TOC were measured using a Hach DR3900 spectrophotometer following Hach Method 10,072 and 10,128, respectively.

Commercially available *A. awamori* (ATCC 22342) and *A. oryzae* (ATCC 46249) were obtained from the American Type Culture Collection (ATCC, Manassas, VA, USA). The fungi cultivation medium was prepared by diluting the hydrolysate with deionized water to achieve a total sugar concentration of 24 g/L. Yeast extract (1 g/L) and a calculated amount of ammonium chloride were added to reach an initial carbon-to-nitrogen (C/N) ratio of 15. The medium was pasteurized at 80°C for three hours in a water bath using sealed bottles to prevent evaporation and then cooled to room temperature before being used for fungal cultivation.

*A. awamori* and *A. oryzae* spores, which had been cultured for four days on potato dextrose agar plates, were inoculated in duplicate into 250 mL flasks containing 100 mL of the prepared medium at a concentration of 10^3^ spores/mL. The use of 100 mL of medium in 250 mL flasks was selected to maintain a liquid depth suitable for pellet formation and to ensure adequate mixing. Preliminary tests showed that smaller effective volumes (e.g., 50 mL) resulted in poor pellet development. Dissolved oxygen measurements under these conditions remained above 30% during cultivation, suggesting that aeration was not a limiting factor. These flasks were incubated in a shaker incubator at 30°C and 200 rpm for five days. Following cultivation, the biomass was harvested using vacuum filtration on glass fiber filter paper. Total suspended solids (TSS) were quantified by drying the biomass at 105°C to a constant weight, and volatile suspended solids (VSS) were determined after incineration at 550°C, according to standard methods (APHA et al., 2017). Sugar concentrations in the spent media were subsequently analyzed HPLC as described above.

### Statistical analysis

Data are presented as mean $\:\pm\:$ standard deviation. Statistical analysis of the experimental data was conducted using one-way or two-way ANOVA, as appropriate, to assess the effects of various factors such as enzyme type, incubation time and enzyme loadings on the measured outcome. Where significant differences were founded (*p* < 0.05), post-hoc pairwise comparisons were carried out using Tukey multiple comparison test to determine specific group differences. These analyses were performed using GraphPad Prism 9.0 (GraphPad Software, San Diego, CA, USA).

## Results and discussion

### Composition of RAHS

After sugar extraction, 50.50% (d.b.) of the nutrients in almond hulls were extracted and separated, leaving 49.50% (d.b) remained as RAHS. The detailed mass balance of major components before and after extraction was presented in Table 2. Notably, 81.35% of the soluble sugars and ash were extracted and transferred to the liquid extract. Meanwhile, cellulose, hemicellulose, pectin and lignin remained in the RAHS.

The composition of RAHS was shown in Table 3. The total amount of cellulose, hemicellulose and pectin accounted for 35.19% of RAHS. In addition, there were 14.81% of soluble sugars in RAHS, representing sugars that remained dissolved in the liquid absorbed by the RAHS. Together, these components represented a theoretical upper limit of approximately 50% total sugar content (g sugar/g RAHS), including both extractable soluble sugars and hydrolysable structural carbohydrates.

**Table 2:** Mass balance of individual compound in 100 g almond hulls before and after extraction (d.b.)

Compounds	Before Extraction	After Extraction	
		Extracts	Residual Solids
Sugar	39.30	31.97	7.33
Cellulose + hemicellulose	12.50	0.00	12.50
Pectin	4.92	0.00	4.92
Lignin	7.89	0.00	7.89
Ash	13.76	11.19	2.57
Others	21.63	7.34	14.29
Total	100.00	50.50	49.50

**Table 3:** Composition of residual almond hull solids after extraction (d.b.)

Compound	Content (%)
Moisture ^*^	17.88
Total solids^*^	82.12
Sugar	14.81
Acid detergent fiber	31.80
Neutral detergent fiber	41.20
Cellulose + hemicellulose	25.26
Pectin	9.93
Lignin	15.94
Ash	5.19

^*^ Values reported on a wet basis

### Enzyme selection and hydrolysis time determination

**Fig. 2:**
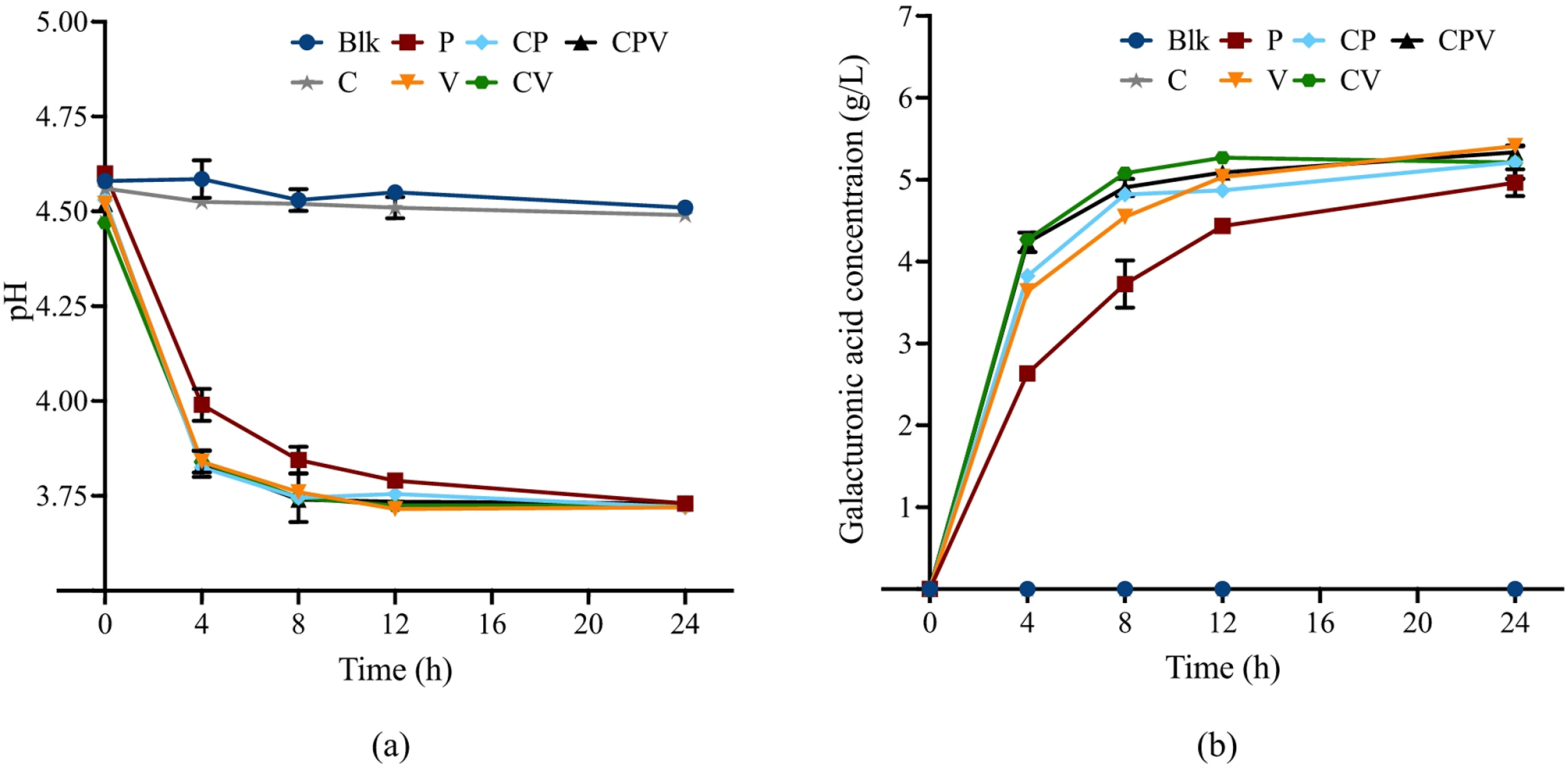
Changes of pH (**a**), and galacturonic acid concentrations (**b**) change during RAHS hydrolysis by different enzymes

The changes of pH during enzymatic hydrolysis of RAHS were shown in Fig. 2a. The pH levels in the blank and C enzyme-only treated groups remained relatively stable, which stabilized around 4.5. In contrast, a rapid pH decline was observed in all groups treated with pectinase enzymes (V and P enzymes), indicating that galacturonic acid generation from pectin hydrolysis was the primary driver of pH reduction. Figure 2b further supported this, where galacturonic acid was detected only in pectinase-treated groups.

According to Kim and Lee (2021) and manufacturer (Novozymes) specifications, the optimal pH ranges for P, V, and C enzymes are 4.0–5.0, 3.3–5.5, and 5.0-5.5, respectively. The pH in all pectinase-treated groups ranged from 3.7 to 4.6, which fell within the optimal range for V enzyme and was close to that for P enzyme. As reported by the manufacturer, the enzyme relative performance of C enzyme at pH 3.7–4.6 was around 50–80% compared to that at optimum pH condition. Usually, a 50 mM sodium acetate buffer is applied to maintain a pH level of 5.0 during enzymatic hydrolysis to optimize conditions (Paz-Cedeno et al. 2022). However, Taniguchi et al. (1977) found that acetic acid, a component of sodium acetate buffer, could inhibit the mycelial growth and sporulation of *A.niger*. Since *A. awamori* is a specific variety of *A. niger*, the addition of sodium acetate buffer was not considered in our procedures to prevent potential inhibition during downstream fungal growth in the hydrolysate.

**Fig. 3:**
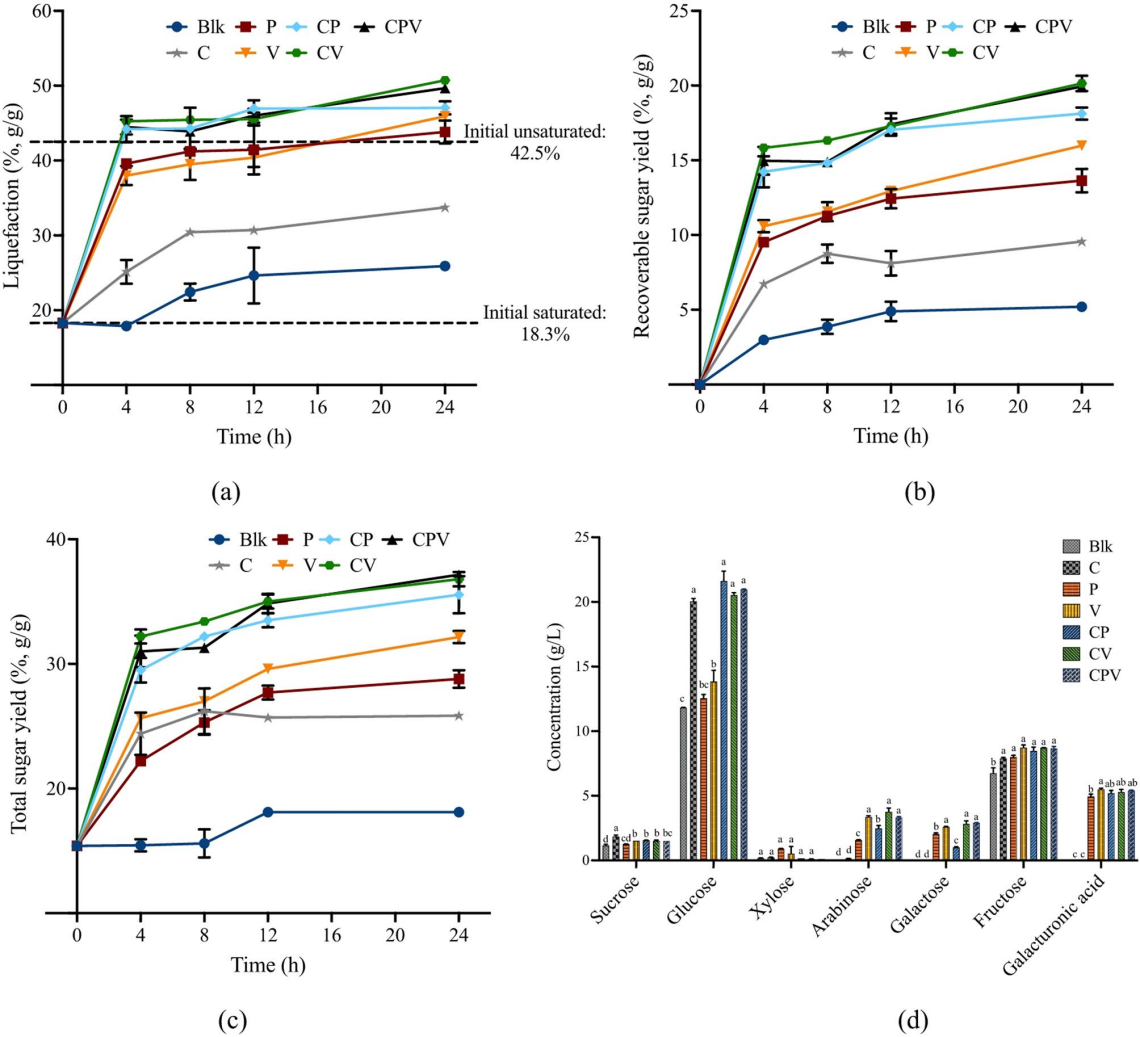
Performance of RAHS hydrolysis by different enzymes. (**a**) Liquefaction efficiency, (**b**) Recoverable sugar yield, (**c**) Total sugar yield from RAHS hydrolyzed by different enzymes, and (**d**) Individual sugar concentrations after 24 h hydrolysis. Bars with different lowercase letters within each sugar group indicated significant differences (*p* < 0.05) as determined by one-way ANOVA followed by Tukey test. Comparisons were conducted separately for each sugar type. Differences across different sugar types were not statistically analyzed

Figure 3a showed that the addition of pectinase (P or V) significantly improved liquefaction efficiency compared to the cellulase alone group or blank. C enzyme is developed primarily for cellulose hydrolysis and contains limited hemicellulase and pectinase activity (Dąbkowska et al. 2017). Within plant cell walls, cellulose is embedded within a complex matrix of hemicellulose, lignin, and pectin, which form a physical barrier that protects cellulose and hinder enzymatic access (Padayachee et al. 2017). Consequently, the effectiveness of C enzyme alone is constrained in pretreated or plant materials with low hemicellulose, pectin or lignin (Kim and Lee 2021). In our study, RAHS contained 15.94% lignin, 9.40% hemicellulose, and 9.93% pectin, indicating that a cellulase-only approach was insufficient for hydrolyzing almond hull fibers. As shown in Fig. 3a, combinations of cellulase and pectinase (CP, CV, and CPV) resulted in higher efficiencies than using cellulase or pectinase alone, demonstrating their synergistic effect. This agrees with findings by Liu et al. (2019), who observed that pretreating apple pomace with pectinase to remove pectin significantly enhanced subsequent cellulose hydrolysis. In addition, Kim and Lee (2021) found that C enzyme alone didn’t enhance protein extraction from plant materials, while both V and P enzyme significantly improved the protein extractability. Since proteins are often embedded near structural carbohydrates, disrupting these complex components can also enhance protein release. Therefore, combining cellulase and pectinase enzymes could potentially increase the nitrogen content in the hydrolysate to support downstream fungal growth.

It should also be noted that at the beginning of hydrolysis, RAHS, which are the solids left after extraction and squeezing, quickly absorbed most of the added water. Initially, water was added to adjust the TS loading. The added water accounted for 42.5% of the total mass, which was defined as the “initial unsaturated baseline” for liquefaction efficiency. Upon mixing with RAHS, the water was quickly absorbed by RAHS, reducing the free water to 18.3%, which was defined as the “initial saturated baseline” for liquefication efficiency. Free water is very important for hydrolysis by facilitating enzymatic reactions, solubilizing hydrolysate and enhancing the mass transfer. Additionally, it helps to reduce the slurry’s viscosity and improves flow properties lowering shear stress needed for mixing (Modenbach and Nokes 2013). With lower amount free water, the mixing and handling of materials become more challenging and energy intensive. Among all the treatments, only the treatments of CP, CV and CPV exceeded the initial unsaturated baseline after just 4 h. Treatments with either P or V enzymes alone required more than 12 h to exceed this baseline. After 24 h, CPV and CV achieved the highest liquefaction efficiency (~ 50%) with no significant difference (*p* > 0.05) between them. A combination of C and V enzymes is recommended for practical application based on both enzyme utilization and hydrolysis performance. Statistical analysis confirmed 24 h was the optimal hydrolysis time for CV treatment. Extending hydrolysis beyond 24 h is not recommended to prevent sugar consumption by microbes and to save operation costs.

Liquefaction efficiency is an important indicator of RAHS breakdown and water release. However, it is the sugar yield that directly quantifies the actual amount of sugars released from the RAHS. Sugar yields, both recoverable and total, were higher in samples treated only with V enzyme than those treated only with P enzyme after 24 h (Fig. 3b and c). One possible reason could be the effect of pH change during hydrolysis, similar to its effect on liquefaction. Additionally, P enzyme primarily contains pectinase, supplemented by $\:{\upbeta\:}$-glucanase and hemicellulase. Its main advantage is in breaking down pectin, which is especially beneficial in fruit and vegetable juice production for clarifying juices (Nguyen and Nguyen 2018). However, V enzyme, which has broader range of carbohydrase activities, enabling it to not only break down pectin but also other structural polysaccharides. In the first eight hours, the total sugar yield from the group treated only with C enzyme was comparable to those treated with V and P. However, the recoverable sugar yield was lower in the C-only group compared to V and P groups. This suggests that while C enzyme facilitated sugar release from RAHS, a substantial portion of these sugars remained dissolved in the liquid within the residual solids. This observation aligned with findings from Holtman et al. (2015), who found that without pectinase, almond hulls retained 70% of the added water, limiting sugar recovery to just 30%. The samples treated by CV exhibited the highest total and recoverable sugar yields after 24 h, with values of 36.80% and 20.15%, respectively, which were comparable to CPV but with lower enzyme usage. The difference between the total and recoverable sugar yield (16.65%) was due to the remaining sugars dissolved in the liquid within the solid phase. This also emphasized the importance of liquefaction in enhancing sugar recovery.

The effect of different enzymes on the hydrolysis was also reflected by the specific sugar concentrations in the hydrolysate (Fig. 3d). Glucose, inherently present in almond hulls, was detected in all treatments and its concentration in blank sample was 11.82 g/L. The release of glucose from RAHS fibers was primarily attributed to the C enzyme. Samples treated solely with the C enzyme exhibited a glucose concentration of 20.06 g/L. The addition of P or V enzymes didn’t significantly increase the glucose concentrations compared to the blank, or any treatments involving C enzyme. In addition to glucose, the hydrolysate also contained a considerable amount of fructose. Since fructose is another sugar naturally present in almond hulls (Cao et al. 2024), it is likely most of it originated from the initial RAHS. As expected, arabinose, galactose, and galacturonic acid were not detected in the blank. The highest concentrations of arabinose and galactose were observed in samples treated with the V enzyme, indicating that the interaction between pectinase and hemicellulase in the V enzyme effectively released these sugars from the pectin’s side chain (Spadoni Andreani and Karboune 2020). None of the enzymes improved the release of xylose. The highest total sugar concentration of 39.87 g/L was achieved from CV treatment, which is sufficient for fungal cultivation without further concentration steps.

In summary, the combination of C and V enzymes proved to be the most effective among the treatments tested and is recommended for future experiments. After 24 h of hydrolysis with CV, pectin conversion reached 66.56% (g/g pectin), the total conversion of hemicellulose and cellulose was 54.83% (g/g hemicellulose + cellulose), and the total fiber conversion was 58.14% (g/g hemicellulose + cellulose + pectin). Further improvements in fiber conversion could potentially be achieved by increasing the loadings of enzymes, which was examined in the following studies.

### Determinations of enzyme loadings

**Fig. 4:**
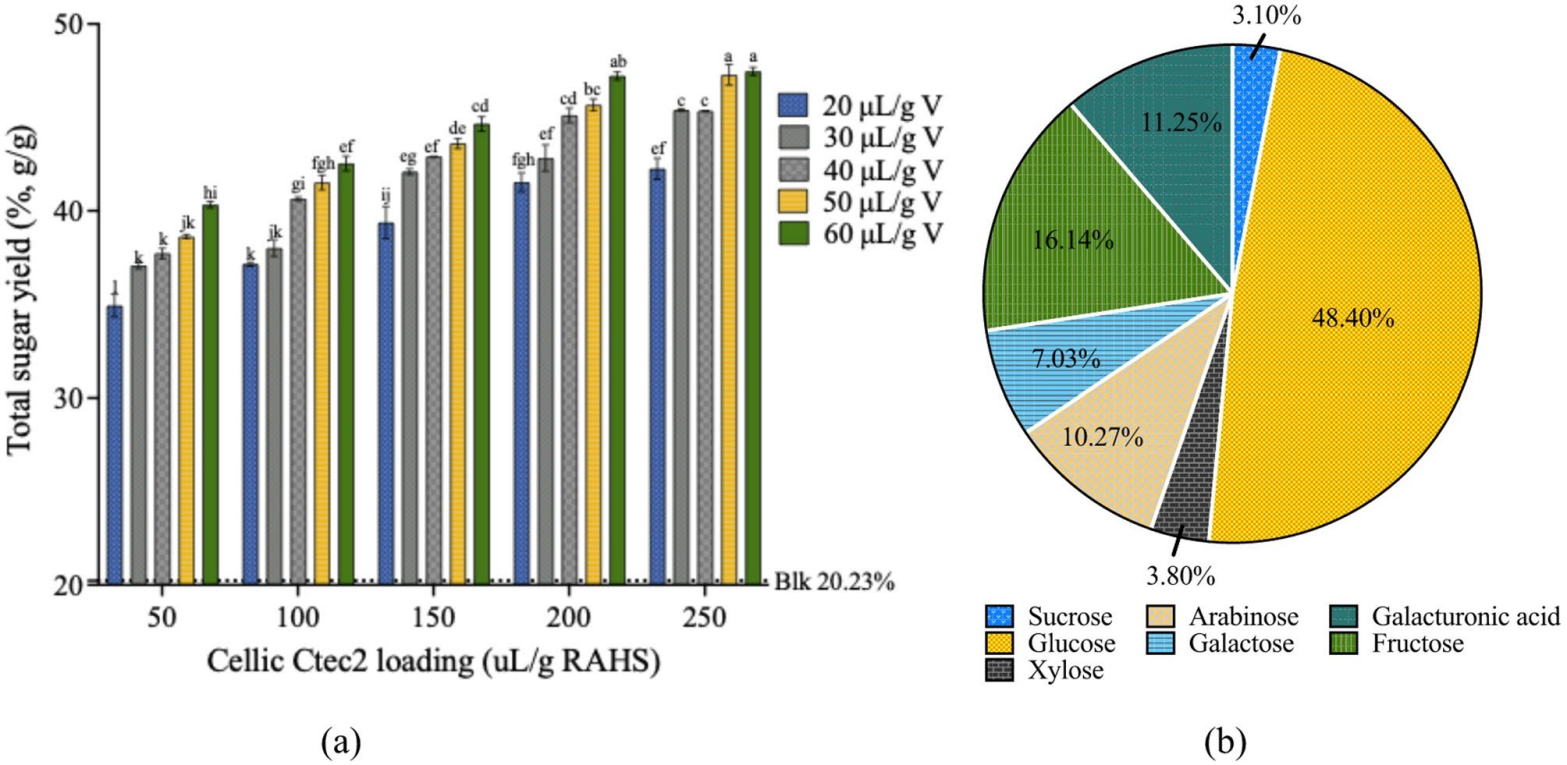
Hydrolysis performance of RAHS. (**a**) Total sugar yield from RAHS hydrolyzed by different enzyme loadings. Different letters indicated a significant difference at *p* < 0.05, and (**b**) Portions of individual sugar yield at optimum conditions (60 $\:{\upmu\:}$L/g RAHS Viscozyme L, 200 $\:{\upmu\:}$L/g RAHS Cellic Cetc2)

The total sugar yields from RAHS after hydrolysis by different enzyme loadings for 24 h were depicted in Fig. 4a. The highest total sugar yield, approximately 47%, was obtained using enzyme combinations of either 200 $\:{\upmu\:}$L/g C mixed with 60 $\:{\upmu\:}$L/g V or 250 $\:{\upmu\:}$L/g C mixed with 50 $\:{\upmu\:}$L/g V or 60 $\:{\upmu\:}$L/g V loadings. To minimize enzyme use, mixing 200 $\:{\upmu\:}$L/g C with 60 $\:{\upmu\:}$L/g V would be recommended. Under this optimal loading, the liquefaction efficiency reached 51.61%, and the recoverable sugar yield was 26.43%. The total sugar concentration reached 56.40 g/L. The breakdown of individual sugar yields under optimal loading was depicted in Fig. 4b. Glucose and fructose were the largest portions, accounting for 48.40% and 16.14% of the total sugar yield, respectively, while galacturonic acid and arabinose also represented significant portions of the total yield, contributing 11.25% and 10.27%, respectively. Understanding the composition of the hydrolysate is important in designing experiments for fungal cultivation.

In conclusion, 88.11% of the total amount of cellulose and hemicellulose and 79.66% of pectin were hydrolyzed, resulting in a total fiber conversion of 86.01% under the optimal enzyme loadings. The enzymatic hydrolysis process yielded 0.47 g sugar/g RAHS (d.b.), including 0.15 g/g from residual soluble sugars. This hydrolysis process enhanced the sugar yield from almond hulls to an additional 0.23 g/g (d.b). By combining extraction and hydrolysis, a total sugar yield of 0.55 g/g (d.b) could be obtained from almond hulls. Comparing to direct enzyme application on raw almond hulls from Sitepu et al. (2023), a sugar yield of 0.56 g/g over three days with the necessity of buffer addition was achieved. However, in their study, galacturonic acid was reported as the most abundant sugars in the almond hulls, almost twice the concentration of glucose, suggesting an unusually high pectin content of over 30%, which was nearly 5 to 10 times higher than the levels reported by other literature (Holtman et al. 2015), the information from Almond Board of California (Almond Board of California 2019), or in our research.

### Large scale enzyme hydrolysis

**Fig. 5:**
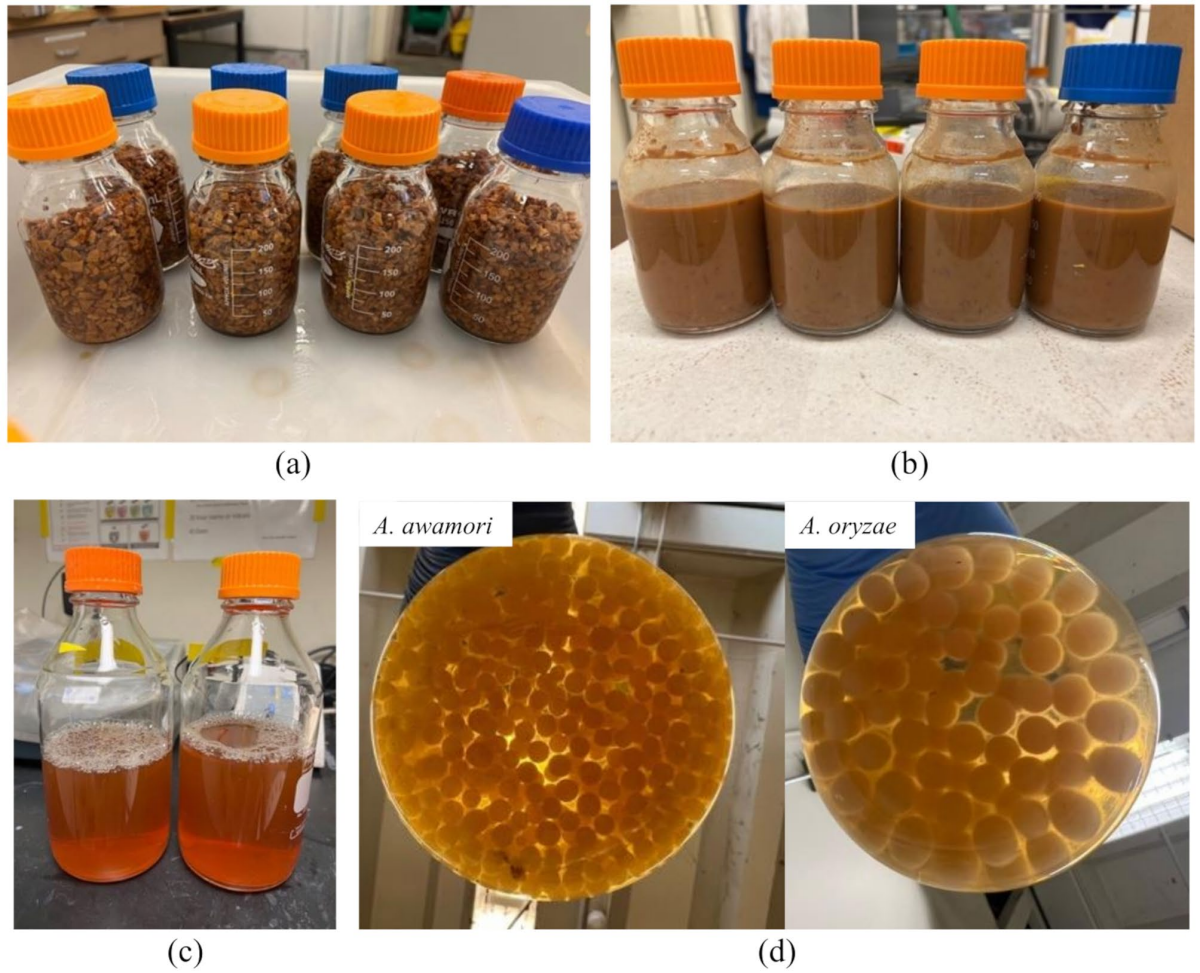
Large scale enzymatic hydrolysis of RAHS for fungal cultivation. (**a**) RAHS in 250 mL glass bottles before hydrolysis, (**b**) RAHS in 250 mL glass bottles after hydrolysis, (**c**) hydrolysate after filtration, (**d**) *A. awamori* and *A.oryzae* pellets grown in RAHS hydrolysate

As determined from previous small-scale studies, a mixture of 200 $\:{\upmu\:}$L/g RAHS of C enzyme and 60 $\:{\upmu\:}$L/g RAHS of V enzyme was selected for large-scale hydrolysis in 250 mL glass bottles. After 24 h of hydrolysis at 50°C, the solid and liquid phases were separated and analyzed. Figure 5a, b, and c depicted how large particles were hydrolyzed into homogeneous slurry-like solids, yielding a clear hydrolysate. The liquefaction efficiency and recoverable sugar yield in glass bottles were higher than those of the small-scale (Table 4). However, total sugar yield and fiber conversion didn’t increase at large scale. A possible explanation for the improved liquefaction efficiency is more effective mixing provided by the glass bottles. The narrow bottom could inhibit the movement and dispersion of solid particles, thereby making mixing less efficient. Conversely, the glass bottles had a wider base which could provide more effective mixing that facilitated better interaction between the enzymes and samples and potentially changed the structure of the samples, leading to the release of more liquid. Research has also demonstrated that the type of reactor and mixing can strongly affected the hydrolysis performance (Du et al. 2014; Olivieri et al. 2021; Palmqvist et al. 2011). These results suggested that mixing is important for hydrolysis. Even though better performance was obtained in large scale bottle experiments, the small-scale tube experiments still provide meaningful conclusions, as they were designed to compare enzyme types, loadings and hydrolysis times under consistent mixing conditions.

The ability to achieve high conversion within 24 h under stable operating conditions supports the scalability of the process. To further reduce production costs, strategies such as enzyme recycling may be explored. As most of the enzyme activity likely remains in the liquid phase following extensive liquefaction, recovery for reuse is technically feasible. We have conducted a techno-economic analysis of the integrated process (Cao 2023). The analysis indicated that the system is cost-competitive with conventional meats and other alternative protein products, supporting its industrial potential. Overall, these findings demonstrate the feasibility of enzymatic hydrolysis of RAHS as an effective strategy for converting agricultural byproducts into value-added fungal biomass.

**Table 4:** Comparison of small- and large- scale enzymatic hydrolysis of RAHS

	Small Scale (20 g)	Large Scale (200 g)
Liquefaction (%, g/g)	51.61 $\:\pm\:\:$ 0.34 ^b^	72.53 $\:\pm\:\:$ 0.56 ^a^
Galacturonic acid concentrations (g/L)	6.40 $\:\pm\:\:$ 0.09 ^a^	5.88 $\:\pm\:\:$ 0.17 ^a^
Galacturonic acid yield (%, g/g RAHS)	5.85 $\:\pm\:\:$ 0.09 ^a^	5.58 $\:\pm\:\:$ 0.01 ^a^
Total other sugar concentration (g/L)	50.00 $\:\pm\:\:$ 0.23 ^a^	47.36 $\:\pm\:\:$ 3.53 ^a^
Total other sugar yield (%, g/g RAHS)	41.36 $\:\pm\:\:$ 0.14 ^a^	41.05 $\:\pm\:\:$ 0.09 ^a^
Total sugar yield (%, g/g RAHS)	47.21 $\:\pm\:\:$ 0.23 ^a^	46.03 $\:\pm\:\:$ 0.10 ^b^
Recoverable sugar yield (%, g/g RAHS)	26.43 $\:\pm\:\:$ 0.05 ^b^	38.61 $\:\pm\:\:$ 0.30 ^a^
Cellulose + hemicellulose conversion (%)	88.11 $\:\pm\:\:$ 0.57 ^a^	86.68 $\:\pm\:\:$ 0.35 ^a^
Pectin conversion (%)	79.66 $\:\pm\:\:$ 1.20 ^a^	75.87 $\:\pm\:\:$ 0.16 ^a^
Total fiber conversion	86.01 $\:\pm\:\:$ 0.75 ^a^	83.63 $\:\pm\:\:$ 0.30 ^a^

Different lowercase superscript letters in the same row indicated significant difference (*p* < 0.05)

### Fungal growth in RAHS hydrolysate

*A. awamori* and *A. oryzae* had different growth performance in RAHS hydrolysate. *A. oryzae* had a significantly higher sugar consumption rate of 81.30%, compared to that (49.09%) for *A. awamori* after 5 days cultivation. Before fungal cultivation, the media was rich in glucose and fructose, which accounted for 64.54% of the total sugar in the media and were the primary carbon sources for most of the fungi growth (Hamad et al. 2014). Although galacturonic acid accounted for 11.25% of the total sugars, its utilization was less preferred compared to other sugars in the media. Even though *A. awamori* is known for pectinase production and can utilize galacturonic acid (de Alencar Guimarães et al. 2022; Dey et al. 2014; Umsza-Guez et al. 2011), it predominantly consumed other sugars first, leaving the galacturonic acid in the hydrolysate unconsumed before harvesting. Extending the cultivation time of *A. awamori* is recommended to improve sugar utilization, and to provide an opportunity for galacturonic acid consumption. In contrast, *A. oryzae* not only consumed 85.01% of the other sugars but also utilized more than half (54.22%) of galacturonic acid within 5 days of cultivation. This demonstrated *A.oryzae*’s capability to utilize galacturonic acid present in the hydrolysate. Compared to the study from Sitepu et al. (2023), which employed a multiphase screening approach try to enable yeast to utilize galacturonic acid from almond hull hydrolysate, none of the 65 yeast strains tested could utilize galacturonic acid, even after adaptions aimed at inducing its consumption. These yeast strains could utilize glucose present in the hydrolysate but failed to consume galacturonic acid even when glucose levels were nearly depleted. In this study, the tested filamentous fungal strains naturally utilized galacturonic acid, highlighting their superior capability to utilize almond hull hydrolysate than yeast.

Both *A. awamori* and *A. oryzae* formed uniform pellets in almond hull hydrolysate and exhibited an orange-yellow color as shown in Fig. 5d. In contrast to their cultivation in almond hull extract, which resulted in brown pellets (Cao et al. 2024). Both strains produced pellets that had a color similar to its corresponding media. This observation suggests that most of the brown pigments were likely removed by water during the sugar extraction process. The VSS content was 0.90 g VSS/g TSS for *A. awamori* and 0.91 g VSS/g TSS for *A. oryzae*. However, *A. oryzae* produced less biomass at 8.54 g TSS/L than that (10.41 g TSS/L) for *A. awamori*. Furthermore, the biomass yield per gram of consumed sugar was also much higher for *A. awamori* at 0.89 g TSS/g sugar, nearly double that (0.43 g TSS/g sugar) of *A. oryzae.* These differences suggest that while *A. oryzae* may utilize the available sugars more rapidly, *A. awamori* is more efficient in converting the consumed sugars into biomass. Another possibility is that more fungal biomass was autolyzed at the center of the *A. oryzae* pellets than in *A. awamori*, as the *A. oryzae* pellets were larger than those of *A. awamori*. Compared to the yeast biomass yield (0.56 g biomass/g sugar) from almond hull hydrolysate reported by Sitepu et al. (2023), A. *awamori* exhibited a higher yield, while *A. oryzae* had a lower yield. A higher biomass yield for *A. oryzae* was also reported by Souza Filho et al. (2018), who achieved 0.57 g biomass/g substrate using pea-processing byproducts primarily composed of starch and protein. In the future, cultivating *A. oryzae* pellets in smaller size can be tested to avoid the autolysis in the center, which could probably increase the biomass yield.

## Conclusions

This study demonstrated the feasibility of converting residual almond hull solids (RAHS) into fungal biomass through enzymatic hydrolysis followed by *A. awamori* and *A. oryzae* cultivation. RAHS contained 35.19% (d.b.) total amount of cellulose, hemicellulose and pectin and 14.81% (d.b.) soluble sugars, which remained after the extraction process, making it a potential feedstock for further sugar recovery and fungal cultivation. By selecting and optimizing different enzymes and loadings, 200 $\:{\upmu\:}$L /g RAHS of Cellic Ctec2 combined with 60 $\:{\upmu\:}$L /g RAHS of Viscozyme L yielded the highest total fiber conversion of 86.01% and liquefaction of 51.61% after 24 h of hydrolysis in 50 ml Falcon tubes. Using 200 ml bottles, liquefaction efficiency improved to 72.53% due to better mixing, while sugar yield remained relative stable (46.03%). Both *A. awamori* and *A. oryzae* were able to grow well in RAHS hydrolysate, forming uniform pellets. *A. oryzae* showed a higher total sugar consumption rate (81.30%) and effectively utilized 54.22% of the galacturonic acid within 5 days cultivation. While *A. awamori* reached higher biomass concentration of 10.41 g TSS/L. The biomass yield of *A. awamori* and *A. oryzae* were 0.89 gTSS/g sugar and 0.43 gTSS/g sugar, respectively. Both fungi preferred other sugars over galacturonic acid. These findings underline the significant potential of RAHS as a substrate for fungal biomass production.

## Supplementary Information

Below is the link to the electronic supplementary material.

Supplementary Material 1

## Data Availability

The data are available from the corresponding author upon suitable request.

## Abbreviations

*A. awamori*: *Aspergillus awamori*
ADF: Acid detergent fiber
*A. oryzae*: *Aspergillus oryzae*
C/N: Carbon-to-nitrogen
C enzyme: Cellic CTec2 enzyme
CP: Mixture of Cellic CTec2 and Pectinex Ultra SPL enzymes
CPV: Mixture of Cellic CTec2, Pectinex Ultra SPL and Viscozyme L enzymes
CV: C enzyme: mixture of Cellic CTec2 and Viscozyme L enzymes
C enzyme: Cellic CTec2:d.b.:dry basis
FBGU/mL: Fungal Beta- Glucanase per mL, FPU/mL: filter paper unit per mL
HPLC: High-performance liquid chromatography
NDF: Neutral detergent fiber
PDA: Photodiode array
P enzyme: Pectinex Ultra SPL enzyme
RAHS: Residual almond hull solids
SCP: Single cell protein
TN: Total nitrogen
TOC: Total organic carbon
TS: Total solids
TSS: Total suspended solids
V enzyme: Viscozyme L enzyme
VSS: Volatile suspended solids
