# Supplementary figures and images for: Enzymatic hydrolysis of almond hulls for cultivation of edible filamentous fungi
Source: Bioresour Bioprocess. 2025 Sep 29;12(1):107. doi: 10.1186/s40643-025-00940-2 (PMC12477097; doi:10.1186/s40643-025-00940-2)

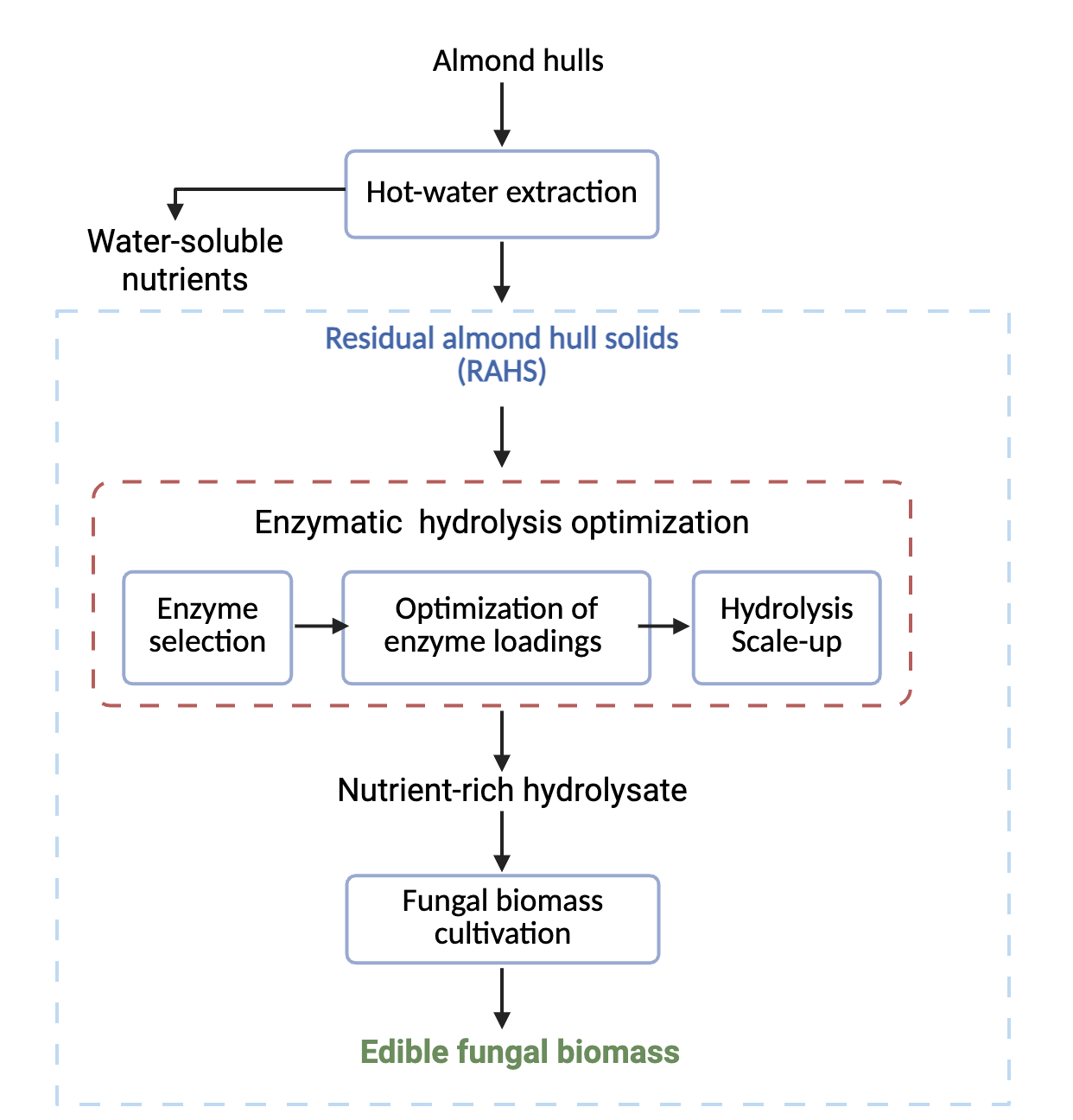

Supplement: Supplementary file 1 — Supplementary Material 1 [file 40643_2025_940_MOESM1_ESM.png]
